# Effect of Follicular Fluid and Platelet-Activating Factor on Lactate Dehydrogenase C Expression in Human Asthenozoospermic Samples

**Published:** 2014-01

**Authors:** Tahereh Esmaeilpour, Mohmmad-Reza Zarei, Soghra Bahmanpour, Elham Aliabadi, Ahmad Hosseini, Mansooreh Jaberipour

**Affiliations:** 1 Department of Anatomy, School of Medicine, Shiraz University of Medical Sciences, Shiraz, Iran;; 2 Shiraz Institute for Cancer Research, School of Medicine, Shiraz University of Medical Sciences, Shiraz, Iran

**Keywords:** Follicular fluid, Platelet-activating factor, LDH-C, Sperm motility, Asthenozoospermia

## Abstract

**Background: **Application of follicular fluid (FF) and platelet-activating factor (PAF) in artificial insemination improves sperm motility. Lactate dehydrogenase C (LDH-C) is a key enzyme for sperm motility. In this study, the effects of FF and PAF on the sperm motility index and LDH-C expression were investigated. Moreover, LDH-C expression was compared between asthenozoospermic and normozoospermic samples.

**Methods: **The expression of LDH-C was examined by quantitative real-time polymerase chain reaction (q-RT PCR) and western blotting after it was treated with optimized concentrations of FF and PAF in twenty asthenozoospermic samples. Also, LDH-C expression was evaluated in five normozoospermic samples.

**Results:** Samples with 75% FF and 100 nM of PAF had an increase in their percentages of progressive and slowly motile sperms and a decrease in their percentages of non-progressive and non-motile sperms. Moreover, LDH-C mRNA transcripts were not changed following PAF and FF treatment, and LDH-C protein was detected in highly progressive motile specimens treated with FF in the asthenozoospermic samples. Furthermore, LDH-C expression was more detectable in the normal sperms.

**Conclusion:** Our results indicated that PAF had more beneficial effects than FF on sperm motility in the asthenozoospermic samples (P=0.0001), although the LDH-C expressions of the sperms were not changed significantly in both groups. We found no association between LDH-C expression and sperm motility after FF and PAF actions. This finding, however, requires further investigation. The fact that LDH-C protein was detected in the normozoospermic, but not asthenozoospermic, samples could be cited as a reason for the infertility in these patients.

## Introduction

Infertility is a worldwide reproductive health problem which affects both men and women with almost equal frequency.^1^^,^^2^ In general, male infertility is due to low sperm counts, poor sperm quality, or both. Genetic defects, hormonal imbalance, and also anatomical problems might cause male infertility in some cases.^2^ Some men are infertile because of poor sperm motility, known as asthenozoospermia. Sperm motility is graded as progressive motility, non-progressive motility, and immotile according to the World Health Organization (WHO) manual criteria.^3^

Sperm motility is considered one of the principal factors in efforts aimed at improving the outcome of assisted reproductive technology.^4^ Some compounds such as follicular fluid (FF), platelet-activating factor (PAF), caffeine, and Pentoxifylline are known to exert stimulatory effects on sperm motility.^5^ Caffeine and Pentoxifylline can penetrate into sperms and trigger some genetic mutation; therefore, the application of these agents is restricted.^6^ FF is a liquid which fills the follicular antrum and surrounds the ovum in an ovarian follicle. It is composed of multiple compounds which can promote sperm motility.^7^ Recently, FF was reported as an important lipid mediator in the organism, capable of stimulating sperm motility and progression.^2^ Although the exact mechanism of FF is uncertain, its role in normal fertility is of considerable significance. C16-PAF, detected in human semen, has greater levels in fertile men than in their infertile counterparts. PAF clearly plays a significant role in reproductive physiology inasmuch as it enhances sperm motility, capacity, and acrosomal reaction.^2^^,^^8^

Sperms produce ATP through glycolysis and aerobic respiration. Sperm mitochondria possess several enzymes or isozymes, including lactate dehydrogenase C (LDH-C),^1^^,^^9^ which contribute significantly to energy production and sperm motility.^10^ Lactate dehydrogenase enzyme is composed of three types: A, B, and C, all of which are detectable in all tissues. This demonstrates the metabolic importance of this molecule in cells.^11^ Also, LDH-C is found in spermatogenetic cells and a lack of it leads to a reduction in progressive sperm motility.^12^

The goal of this study was to probe into potential links between the stimulating effects of FF and PAF as effective agents and LDH-C as an effective gene on sperm motility. We also compared LDH-C expression between asthenozoospermic and normozoospermic cases. Knowledge about the molecular mechanisms involved in sperm motility and relevant genes could usher in new therapies for infertility or, in contrast, contraceptive methods via biotechnological methods. 

## Materials and Methods

*Semen Samples and Experimental Design*

Semen samples were obtained from idiopathic asthenozoospermic (n=20) and normozoospermic (n=5) donors collected by masturbation after 2-4 days of sexual abstinence between July 2011 and December 2011 at Shiraz Infertility Center. The samples were allowed to liquefy at 37°C, for up to 30 min and analyzed according to the WHO guidelines.^3^ Infectious sperms and samples with >1×10^ 6^ round cells/ml were excluded. The Ethics Committee of Shiraz University of Medical Sciences approved the use of the volunteers’ semen for the present study. This investigation was designed as an experimental study. To rule out the possibility of any contamination by residual cells (germ cells or polynuclear cells), the samples were prepared by two-layer (40:80) AllGrade (LifeGlobal, USA) gradient centrifugation at 400´g according to the manufacturer’s instruction. Depletion of germ cells and leukocytes was confirmed by light microscopy and c-kit expression.

*Acquisition of Follicular Fluid*

Human FF was collected during oocyte retrieval from women (n=5) participating in an in vitro fertilization (IVF) program. Only FF with no blood contamination from mature follicles was used in this study. The FFs obtained were centrifuged in 1000×g for 20 min and filtered through 0.2 µm membranes (Millipore Corp., Bedford, MA, USA) to remove cells and cell debris. They were thereafter pooled and stored in -70ºC until further tests.

*Optimization of Follicular Fluid and Platelet-Activating Factor*

Exposure time of the concentrations of FF and PAF (Sigma-Aldrich, Steinheim, Germany) was optimized in the asthenozoospermic samples. Various concentrations of the pooled FF in Ham’s F10 media (Sigma-Aldrich) (v/v), including 0, 25%, 50%, 75%, and 100%, and different concentrations of PAF in Ham’s F10, including 0, 10, 100, and 1000 nM, were incubated with the sperms for 0, 1, 2, and 4 h. The sperms were harvested and their motility was examined by light microscopy.

*Sample Groups and Motility Index Analysis*

The washed semen was divided into three groups: the first group was cultured in Ham’s F10 media for 2 h as the control group and the second and third groups were treated with 75% FF and 100 nM of PAF (Sigma-Aldrich) for 2 h, respectively, as the experimental groups. The sperm motility index was assessed and classified as progressive (rapid, slow, and total) and non-progressive. Immotile sperm was also considered for the analysis. Sperm motility was manually assessed by a single skilled individual in duplicate before and after FF and PAF treatment. 

*Quantitative real-Time Polymerase Chain Reaction* (q RT-PCR)**

The total RNA of the control and the two experimental groups with FF or PAF were extracted using the Biozol^®^ RNA isolation reagent (BioFlux, Tokyo, Japan), according to the manufacturer’s protocol. The purity of the RNA samples was determined by UV spectrophotometry at 260 / 280 nm. Total RNA was reverse-transcripted by RevertAid^TM^ First Strand cDNA Synthesis (Fermentas, Finland), as is described by the manufacturer. Specific primers and TaqMan probes were designed^13^ for 18s rRNA (as housekeeping gene), LDH-C, and c-kit (table 1). The expressions of the genes were determined by quantitative real-time polymerase chain reaction (qRT-PCR) choromo-4 detector system (BioRad, USA). Gene expression was calculated based on the 2^–ΔCt^ method in each condition (following formula).^14^

**Table 1:** Sequences of specific primers and TaqMan probes

**Sequences (5´→3´)**	**Target gene**	
CGAACGTCTGCCCTATCAACTT	Forward	18s rRNA
ACCCGTGGTCACCATGGTA	Reverse	
ACACACCGCCCGGTCCTACTACCG	Probe (5´; FAM, 3´; TAMRA)	
ACTCTGCCCGTTTCCGTTACCT	Forward	LDH-C
CCCCACTCCATAAGGGCACACT	Reverse	
TGGGTGTCCACCCCACAAGCTGCCA	Probe (5´; FAM, 3´; TAMRA)	
GCATTCAAGCACAATGGCACGGT	Forward	c-kit
GGGTGTGGGGATGGATTTGCTCTTT	Reverse	
ACAACGATGTGGGCAAGACTTCTGCCT	Probe (5´; FAM, 3´; TAMRA)	

The result of the gene expression study revealed that c-kit was not expressed in our samples, showing that washing sperms with AllGrade excluded other cells such as germ cells in the specimens. 

Gene expression in each condition=2^–ΔCt^=2^– (Ct LDH-C–Ct 18s rRNA)^

*Western Blotting*

Sperm proteins were extracted in two steps. First, the sperms were denatured in TCA buffer (50 mg of trichloroacetic acid, 0.5 ml 2-mercaptoethanol, and 50 ml acetone), incubated overnight at 4ºC, and centrifuged. Then, the sperm protein pellets were dissolved in lysis buffer (0.2% CHAPS, 0.1%DDT, and 5M Urea). The sperm protein concentrations were determined by Bradford assay. Before LDH-C expression analysis, 10µg of each sample was pooled based on its primary progressive motility index. The sperm proteins were separated on 12% SDS-PAGE and blotted overnight into PVDF membrane. The membranes were blocked by 5% skimmed milk and stained overnight at 4ºC by HRP-conjugated anti LDH-C (ab7639, Abcam, USA) and HRP-conjugated anti β-actin (ab20272, Abcam, USA) as positive controls. The presence of LDH-C and β-actin was visualized by chemiluminescent substrate (Thermo Fisher Scientific, USA) and developed in radiographs.

*Statistical Analysis*

The data were analyzed by SPSS software (version 15.0). The paired T test and the Wilcoxon matched pair test were utilized to compare the sperm motility index and gene expression between the groups, respectively. P<0.025 (CI=95%) was considered as statistically significant. 

Graphs were plotted using GraphPad Prism 5.0. 

## Results

*Optimization the Follicular Fluid and Platelet-Activating Factor Concentrations*

**Figure 1:**
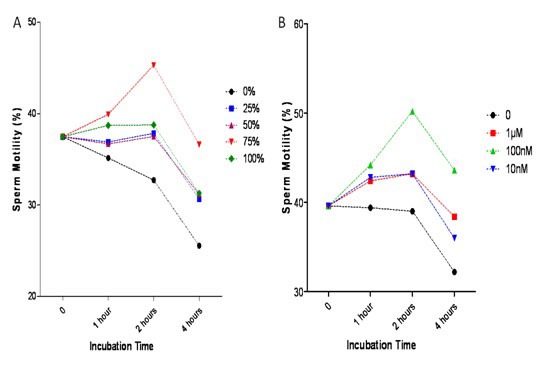
Optimization of follicular fluid (FF) (A) and platelet-activating factor (PAF) (B) concentrations. Various concentrations of FF (0%, 25%, 50%, 75%, and 100%) and PAF (0, 10, 100, and 1000 nM) in Ham’s F 10 media were incubated with sperms for 0, 1, 2, and 4 hours. Sperm motility was determined by light microscopy. Sperm motility was significantly increased in 75% FF incubated for 2 hours and 100nM of PAF incubated for 2 and 4 hours

The semen samples were treated with various concentrations of FF (0, 25%, 50%, 75%, and 100%) and PAF (0, 10, 100, and 1000 nM) in Ham’s F10 media for 0, 1, 2, and 4 h. Figure 1 shows that 75% FF (A) and 100 nM of PAF (B) for 2 h in the culture media had the best effect on the sperm motility rate.

*Effect of Follicular Fluid and Platelet-Activating Factor Treatment on Sperm Motility Index*

**Figure 2:**
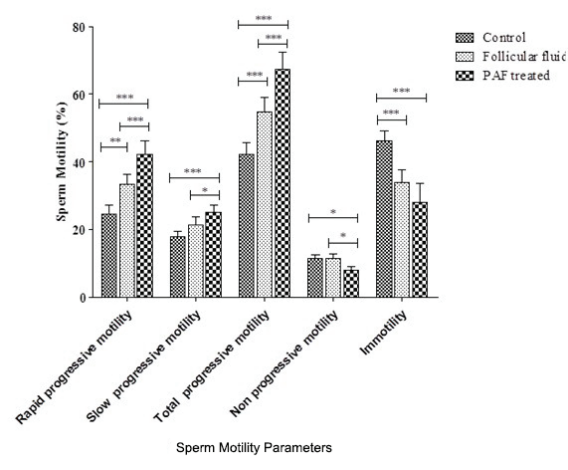
Graph shows the percentage of sperm motility after treatment with 75% follicular fluid (FF) and 100nM of platelet-activating factor (PAF). Data were analyzed by Wilcoxon matched pair test (*P<0.05; **P<0.01; ***P<0.001).

The sperm motility index has been summarized in Figure 2. The percentage of the sperms with highly progressive motility significantly increased in comparison with the control group (24.4%) after FF and PAF treatments (33.2% and 42.1%, respectively; P=0.003 and P=0.005). The percentage of the sperms with slow progressive motility was slightly increased compared to the control group (17.95% to 21.4% in FF treatment; P=0.12) and (17.95% to 25.1% in PAF treatment; P=0.004). There was no difference between the mean percentage of non-progressive sperms between the control and FF treatment groups (11.5%), but PAF led to a significant decrease in non-progressive sperm populations (8.05%; P=0.016). Moreover, the immotile sperm populations were depleted after FF and PAF treatments (33.8%; P=0.0003 and 28.1%; P=0.0001, respectively) compared to the control group (46.1%).

*Effect of Follicular Fluid and Platelet-Activating Factor Treatment on Lactate Dehydrogenase C* *Gene Expression*

**Figure 3:**
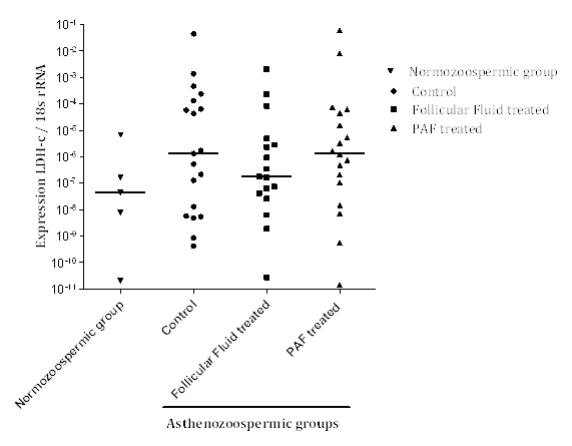
Lactate dehydrogenase C (LDH-C) mRNA expression. Expression of LDH-C transcripts was evaluated by real-time polymerase chain reaction (PCR) in normozoospermic and asthenozoospermic samples after treatment with follicular fluid (FF) and platelet-activating factor (PAF). The median of LDH-C gene expression between the control as well as the FF and PAF-treated groups and normozoospermic groups is demonstrated in the scatter plot by solid line. The expression of LDH-C mRNA was not different between the various groups of this study.

After two hours of treatment with FF and PAF, LDH-C transcripts were evaluated by quantitative real-time PCR. Figure 3 shows that the LDH-C transcript expressions were similar to that in the control group after FF and PAF treatment (P>0.05). The expression of LDH-C was also examined in five normozoospermic samples and similar result was obtained (figure 3).

*Effects of Follicular Fluid and Platelet-Activating Factor Treatment on Lactate Dehydrogenase C* *Protein Expression*

**Figure 4:**
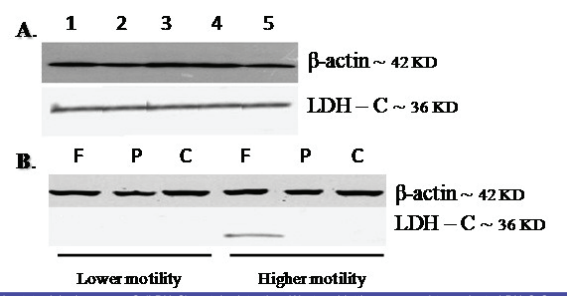
Lactate dehydrogenase C (LDH-C) protein detection. Western blotting was used to evaluate LDH-C C protein in normozoospermic (A) and asthenozoospermic samples (B). All five normoozoospermic samples expressed LDH-C protein, while the asthenozoospermic samples (except for higher motility sperms, which were treated with follicular fluid) did not express LDH-C. F: Follicular fluid-treated sperms; P: Platelet-activating factor-treated sperms; C: Control

The expression of LDH-C protein was also examined after FF and PAF treatment in the normozoospermic and asthenozoospermic samples by western blot analysis (figure 4). All the five normozoospermic sperm samples showed LDH-C protein expression (figure 4A). Only the pools of the asthenozoospermic samples with higher motility and lower motility were used to detect LDH-C protein. While LDH-C protein expression was detected only in the FF-treated sperms with high motility, LDH-C expression was not detectable in the other asthenozoospermic samples (figure 3B).

## Discussion

Sperm motility is an important predictor of male fertility. Sperms obtain their energy from aerobic respiration and anaerobic glycolysis pathway, which take place in the mitochondria located in the middle segment, and from glycolytic enzymes in the main segment of the flagellum and the surface of the fibrotic membrane, respectively.^15^

Many studies have shown that FF enhances sperm motility, especially progressive motility, in vitro. Various concentrations of FF have been tested at different time points by different investigators.^7^^,^^15^^-^^18^ Mendoza et al.^16^ used 20% FF in B2 medium for 6 h, and Kulin et al.^17^ showed that 100% FF incubated for 6 h improves sperm motility. Getpook et al.^18^ used a range of concentrations (0-100%) of FF at different time points and revealed that the highest improvement of sperm motility can be detected at 20-50% FF. They also reported that over 80% FF inverts its beneficial properties and that sperm incubation with FF more than 12 h reduces sperm motility.

The present study confirms the results of the Getpook et al.^18^ study, which reported that FF increases sperm progressive motility in a time and dose-dependent manner. Sperm treated with 75% FF for 2 hours significantly increased the percentage of progressive motile sperms and decreased the percentage of non-motile sperms, but the percentage of slow progressive and non-progressive sperms did not alter significantly. Unlike the current study, most studies have utilized normal specimens with good quality, which can explain the disparity in results. Briton-Jones et al.^19^ incubated oligospermic and normal samples with FF for 2 hours, and detected an increase only in the percentage of progressive motility in the normal specimen. Because of its various components, FF can affect sperm motility. Hamamah et al.^20^ demonstrated that FF changes the sperm cell membrane and consequently, sperm motility.

In the current study, washed sperms of asthenozoospermic patients were also treated with various concentrations of PAF at different time points. The results showed that 100 nM of PAF with 2 hours’ incubation had the highest effect in terms of promoting sperm motility. Jarvi et al.^21^ reported that sperm incubation with 0.5-100 nM of PAF leads to an increase in linear motility, with the best effect obtained from 50 nM of PAF. Another investigation reported that PAF can enhance the sperm motility of normal sperms at a concentration of 0.1 μM for 15 minutes and improve the sperm motility of poor motile sperms at 0.5 μM with 60 minutes’ incubation.^22^

The results of the present experiment indicated that PAF, similar to FF, statistically increased the rapid and slow progressive percentages of sperm motility in comparison with the control ones, and also, PAF led to a significant decrease in the non-progressive and non-motile sperm populations. Our results chime in with those reported by a large number of studies. Sengoku K. et al.^23^ showed that PAF can improve human sperm functions, especially sperm motility, and that the application of PAF receptor antagonist has reverse effects on sperms. The positive effects of PAF on the motility of freshly isolated and frozen sperms have also been previously reported.^24^ It has been demonstrated that 10 nM of PAF for 4 hours increases threefold the sperm motility of normal sperms.^25^ Furthermore, it has been shown that PAF, PAF receptor, and related mRNA have lower expression rates in infertile sperm samples in comparison to normal sperms.^19^ This may address the different findings between normal and abnormal sperms. Fabbri et al.^26^ found that FF can affect positively on curved sperm motility and sperm head movement but it can decrease linear sperm motility. In contrast, another study reported that FF is critical for linear sperm motility.^24^ In the present study, sperm motility styles were not assessed; nonetheless, PAF treatment increased sperm motility more than FF treatment.

The exact mechanism whereby PAF can improve sperm motility has to yet to be fully elucidated. Be that as it may, it seems that PAF can induce inositol triphosphate (IP3) and diacylglycerol (DAG) formation and lead to a rise in the intracellular calcium ion level. All these events increase actin membrane network depolymerization and phospholipase activation, both of which can increase cellular movement and sperm motility.^27^

In the present study, LDH-C expression was also investigated by western blotting. The expression of LDH-C protein was observed in the normal samples but not in the asthenozoospermic ones with any treatment. The highly progressive asthenozoospermic sperms treated with FF showed a slight expression of LDH-C, whereas the other asthenozoospermic sperms treated with FF and PAF did not express LDH-C protein. Therefore, it seems that the absence of LDH-C may be deemed one of the causes of infertility in asthenozoospermic patients. It is likely that FF contains some factors that accelerate LDH-C translation in highly progressive asthenozoospermic sperms. Nevertheless, PAF, as one of components of FF, definitely cannot be regarded as one of such factors. These data are inconsistent with those reported in recent years insofar as the latter mentions the sperm as a genetically active cell and refuses the silencing sperm hypothesis.^28^ Unfortunately, as yet there is no literature concerning laboratory investigation on the addition of exogenous FF and PAF to a sperm medium of human spermatozoa for the evaluation of LDH-C protein expression to compare with our study.

Two hypotheses have been presented about protein synthesis in sperms.^29^ Based on the first theory, mRNA translation may be utilized by extra mitochondrial ribosomes and the second theory states that mRNA translation may be performed by intra mitochondrial ribosomes. There is, however, some controversy about mRNA entrance into mitochondria. A large number of studies have confirmed the first theory and shown that that mRNA translation occurs by extra mitochondrial ribosomes.^30^ It is probable that this protein enters mitochondria for additional processing after translation.^31^ The expression of LDH-C was increased after FF treatment in the current study. This protein acts in the anaerobic glycolytic pathway (extra mitochondria); therefore, it may be concluded that LDH-C is synthesized by extra mitochondrial ribosomes.

PAF receptors are located in the middle segment of the sperm tail, while LDH-C protein is positioned in the main segment of the sperm tail.^32^^,^^33^ PAF probably increases sperm motility via mitochondria stimulation.

The expression of LDH-C gene was slightly increased in the FF and PAF sperms of the asthenozoospermic patients, but this change was not statistically significant in comparison to the control group. The level of LDH-C transcripts in the PAF-treated sperms was significantly increased compared to the FF-treated ones; nevertheless, what should be taken into account is that PAF and FF did not promote LDH-C expression and it is likely that other factors participated in this pathway. As FF treatment led to a slight drop in LDH-C mRNA levels and induced LDH-C protein expression, it may be argued that FF probably induced the translation of some LDH-C mRNAs that had been previously transcripted. Furthermore, the level of LDH-C transcripts did not statistically differ between the normozoospermic sperms and the asthenozoospermic ones. This finding does not tally with the Wang et al.^10^ study, which reported a lower expression of LDH-C gene in poor motility samples. This discrepancy can be in consequence of two reasons. Firstly, Wang et al. investigated LDH-C expression in samples with motility below 5%, while our study population had sperm motility of about 23.5±19.9%. Secondly, our study population was smaller than that of the Wang et al. study.

## Conclusion

PAF is more effective than FF in promoting sperm motility. Although LDH-C expression was slightly affected after incubation with PAF and FF, the difference was not significant. Hence, this effect could not be considered the principal factor in LDH-C induction by PAF and FF. Indeed, many other genes may contribute to sperm motility and this is an area that requires further investigation. In addition, while LDH-C protein was detected in the normozoospermic sperms, it was not detectable in the asthenozoospermic sperms: this finding may be underscored as one of the causes of infertility in these patients. Our results also revealed that FF-treated asthenozoospermic sperms expressed slight amounts of LDH-C protein. Thus, some factor in FF, and not PAF, may improve LDH-C translation in asthenozoospermic samples.
